# Novel Mitochondria-Targeted Heat-Soluble Proteins Identified in the Anhydrobiotic Tardigrade Improve Osmotic Tolerance of Human Cells

**DOI:** 10.1371/journal.pone.0118272

**Published:** 2015-02-12

**Authors:** Sae Tanaka, Junko Tanaka, Yoshihiro Miwa, Daiki D. Horikawa, Toshiaki Katayama, Kazuharu Arakawa, Atsushi Toyoda, Takeo Kubo, Takekazu Kunieda

**Affiliations:** 1 Department of Biological Sciences, Graduate School of Science, The University of Tokyo, Hongo 7-3-1, Bunkyo-ku, Tokyo, Japan; 2 Faculty of Medicine, University of Tsukuba, 1-1-1 Ten-noudai, Tsukuba, Japan; 3 Institute for Advanced Biosciences, Keio University, Endo 5322, Fujisawa, Kanagawa, Japan; 4 Database Center for Life Science, 178-4-4 Wakashiba, Kashiwa-shi, Chiba, Japan; 5 Comparative Genomics Laboratory, Center for Information Biology, National institute of genetics, Yata 1111, Mishima, Shizuoka, Japan; 6 Advanced Genomics Center, National institute of genetics, Yata 1111, Mishima, Shizuoka, Japan; Louisiana State University and A & M College, UNITED STATES

## Abstract

Tardigrades are able to tolerate almost complete dehydration through transition to a metabolically inactive state, called “anhydrobiosis”. Late Embryogenesis Abundant (LEA) proteins are heat-soluble proteins involved in the desiccation tolerance of many anhydrobiotic organisms. Tardigrades, *Ramazzottius varieornatus*, however, express predominantly tardigrade-unique heat-soluble proteins: CAHS (Cytoplasmic Abundant Heat Soluble) and SAHS (Secretory Abundant Heat Soluble) proteins, which are secreted or localized in most intracellular compartments, except the mitochondria. Although mitochondrial integrity is crucial to ensure cellular survival, protective molecules for mitochondria have remained elusive. Here, we identified two novel mitochondrial heat-soluble proteins, RvLEAM and MAHS (Mitochondrial Abundant Heat Soluble), as potent mitochondrial protectants from *Ramazzottius varieornatus*. RvLEAM is a group3 LEA protein and immunohistochemistry confirmed its mitochondrial localization in tardigrade cells. MAHS-green fluorescent protein fusion protein localized in human mitochondria and was heat-soluble *in vitro*, though no sequence similarity with other known proteins was found, and one region was conserved among tardigrades. Furthermore, we demonstrated that RvLEAM protein as well as MAHS protein improved the hyperosmotic tolerance of human cells. The findings of the present study revealed that tardigrade mitochondria contain at least two types of heat-soluble proteins that might have protective roles in water-deficient environments.

## Introduction

Tardigrades are microscopic invertebrates comprising the phylum Tardigrada. Although all tardigrade species are primarily aquatic and require water to grow and reproduce offspring, some terrestrial species can tolerate almost complete desiccation by entering an ametabolic dehydrated state, referred to as anhydrobiosis [1]. Dehydrated tardigrades exhibit extreme tolerance against various environmental stresses [2,3]. The molecular mechanism of anhydrobiosis in tardigrades, however, remains largely unknown.

Anhydrobiotic ability is observed in selected invertebrate species belonging to a few animal phyla, such as tardigrades, nematodes, arthropods, and rotifers. Trehalose accumulates extensively in nematodes and arthropods in a dehydrated state, and is suggested to play an important role in their anhydrobiotic ability [4–6]. In contrast, low or no trehalose accumulation is detected in tardigrades and rotifers, suggesting that anhydrobiosis is achieved by another mechanism [7,8].

Another candidate molecule is late embryogenesis abundant (LEA) protein. LEA protein was originally detected as an abundantly accumulated protein in desiccating plant seeds and later in various anhydrobiotic animals in the phyla mentioned above [9]. In principle, LEA protein expression is significantly induced by desiccation in most anhydrobiotic animals [10,11]. LEA proteins are highly hydrophilic and intrinsically unstructured proteins [12], and maintain solubility even after boiling in water (heat-soluble) [11,13]. *In vitro* studies demonstrated that LEA proteins weakly interact with other macromolecules to prevent undesirable aggregation of proteins [14,15] or conformational changes of lipid membranes by acting as a ‘molecular-shield’ [15]. LEA proteins are also proposed to act as ion-scavengers and anti-oxidants, but their precise functions are not completely understood. Consistent with their protective activity *in vitro*, introduction of LEA proteins to stress-sensitive cells like human cells improves tolerability against various water stresses, such as dehydration, hyperosmotic treatment, and freezing [16–18]. Several LEA proteins potentially localize in specific subcellular compartments, like mitochondria, cytosol, or endoplasmic reticulum, and likely protect macromolecules in the respective compartments [16–19].

Although LEA-like sequences are found in EST or TSA databases of tardigrades [20–22], our previous heat-soluble proteome study revealed that two novel protein families other than LEA proteins are the predominant heat-soluble proteins in the anhydrobiotic tardigrade *Ramazzottius varieornatus*: cytoplasmic abundant heat soluble (CAHS) proteins and secretory abundant heat soluble (SAHS) proteins [23]. CAHS proteins localize in the cytoplasm and occasionally in the nucleus, whereas SAHS proteins are mainly secreted to the extracellular space and a minor population is detected in secretory organelles. Subcellular localization analyses of these proteins using green fluorescent protein (GFP)-fused protein in culture cells clearly demonstrated that mitochondria lack both of these heat-soluble proteins [23]. Other potent heat-soluble proteins like LEA proteins or potential protective molecules have not yet been identified in tardigrade mitochondria.

In the present study, we identified two novel mitochondrial heat-soluble proteins from the anhydrobiotic tardigrade *R. varieornatus*. One of the identified proteins, RvLEAM, is the first mitochondrial LEA protein identified in tardigrades. The other identified protein, Mitochondrial Abundant Heat Soluble (MAHS) protein, is not an LEA protein, and rather forms a novel mitochondrial heat-soluble protein family that is conserved in tardigrades, but not found in other phyla. Furthermore, we demonstrated that either RvLEAM or MAHS protein could confer hyperosmotic tolerance to human cells. The presence of MAHS protein, together with previously identified CAHS and SAHS proteins, indicates that tardigrades possess a unique repertoire of heat-soluble proteins covering most cellular compartments, including the mitochondria. The identified repertoire of tardigrade-unique heat-soluble proteins will provide important clues to the desiccation tolerant mechanism in tardigrades.

## Materials and Methods

### Animals

For all experiments, YOKOZUNA-1 strain *R. varieornatus* was used. The strain was established from a single individual in a previous study [24] and was maintained on water-layered agar plates by feeding algae, as previously described [24]. Tardigrades lay eggs at intervals of approximately 4 to 5 days. Embryos with eggshells were collected at multiple developmental stages and collectively subjected to immunohistochemical analyses.

### Sequence analysis

Three independent programs, Wolf PSORT [25], TargetP [26,27], and MitoProt2 [28], were used to predict the subcellular localization of candidate proteins. The PORTER program was used to predict the secondary structures of RvLEAM (LC002821) and MAHS (LC002822) [29]. Similarity searches were performed by BLASTP or TBLASTN using NCBI non-redundant (nr), EST and TSA databases. A Kyte-Doolittle hydropathy plot was analysed with Genome Consortium for Active Teaching Genomics Tools (http://gcat.davidson.edu/DGPB/kd/KD.html) and a grand average of hydropathy (GRAVY) score was calculated by Sequence Manipulation Suite Protein GRAVY (http://www.bioinformatics.org/sms2/protein_gravy.html) [30]. Motif search and de-novo motif discovery were performed by InterProScan (http://www.ebi.ac.uk/interpro/) [31] and MEME program (http://meme.nbcr.net/meme/cgi-bin/meme.cgi) [32], respectively. Multiple alignments and Sequence Logo analysis were performed using Clustalx and CLC Main Workbench 6 (CLC bio).

### Subcellular localization of GFP-fused protein

Expression of GFP-fused protein and analyses of its subcellular localization were performed essentially as described previously [23]. Briefly, expression constructs were prepared by inserting coding sequences of candidate proteins into the pAcGFP1-N1 vector (Clontech) to fuse GFP at the C-terminus of target proteins. The constructs were transfected to human HEp-2 or HEK293T cells using X-tremeGENE 9 reagent (Roche). The parental cell lines were provided by the RIKEN BRC through the National Bio-Resource Project of the MEXT, Japan. After 24-h incubation, nuclear DNA and mitochondria were stained with Hoechst 33342 (Lonza) and MitoTracker Red CMXRos (Lonza), respectively. Fluorescent images were obtained using a confocal microscope, LSM710 (Carl Zeiss).

### Immunohistochemistry

Collected embryos were fixed with 4% paraformaldehyde in phosphate-buffered saline (PBS) overnight. After rinsing the samples with PBS, they were incubated overnight in 30% sucrose in PBS and embedded in 2% agar block. The ager blocks were trimmed and embedded in O.C.T compound (Tissue-Tek). Frozen sections were cut at a 10-μm thickness using a cryostat and collected on glass slides. The slides were rinsed with 0.1% Tween-20 in Tris-buffered saline (TBS) and microwaved in 0.1 M sodium citrate buffer (pH 6.0) for antigen activation. Anti-RvLEAM rabbit antiserum was raised against bacterially expressed and purified full-length RvLEAM protein. To visualize the mitochondria, we used anti-ATP5A [15H4C4] antibody (Abcam). After incubation with blocking buffer (2% goat serum and 0.1% Tween-20 in TBS), anti-ATP5A mouse antibody solution was added at a dilution of 1/250. After three washes with 0.1% Tween-20 in TBS, the specimens were reacted with secondary anti-mouse IgG antibody labelled with Alexa-Fluor 568 in blocking buffer for 1 h at room temperature. After three washes with 0.1% Tween-20 in TBS, anti-RvLEAM antiserum solution was added at a dilution of 1/500. After three washes with 0.1% Tween-20 in TBS, the specimens were reacted with secondary anti-rabbit IgG antibody labelled with Alexa-Fluor 488 in blocking buffer for 1 h at room temperature. Prior to observation with a confocal microscope, DNA was stained with Hoechst 33342 (Lonza).

### Heat-solubility assay

The heat-solubility assay was performed as described previously [23] with slight modification. Recombinant proteins tagged with 6xHis at the N-terminus were expressed in *Escherichia coli* BL21(DE3) using the pET system (Novagen) for RvLEAM and MAHS, and the pCold-I system (Takara) for ATPM1 (Actively transcribed Transcript encoding Potential Mitochondrial 1, LC002823). Predicted mitochondria-targeting peptides were not included in the construct for MAHS and ATPM1. After inducing proteins according to the manufacturer’s protocol, the bacterial pellets were lysed by sonication in PBS containing protease inhibiter cocktail, Complete EDTA-free (Roche). His-tagged proteins were captured with Ni-NTA Superflow (Qiagen) and eluted with 250 mM imidazole in PBS after extensive washes with PBS. Imidazole was removed by dialysis against PBS using a micro-dialyzer, TOR-14K (Nippon Genetics). Prior to heat treatment, the protein solutions were centrifuged at 10,000 rpm for 30 min at 4°C and supernatants were used as a starting material for the heat-solubility assay. After heat treatment at 92°C for 15 min, samples were cooled to room temperature and centrifuged at 10,000 rpm for 30 min to separate the supernatants and precipitates as heat-soluble fractions and insoluble fractions, respectively. Protein fractions were analysed by sodium dodecyl sulphate-polyacrylamide gel electrophoresis with Coomassie Brilliant Blue staining.

### Osmotic tolerance assay

We utilized an Epstein-Barr virus-based vector system to efficiently establish stably transfected cells [33]. HEp-2 cells were used as parental cells and were essentially maintained in Eagle’s minimal essential medium (EMEM; 286–317 mOsm/kg; Sigma-Aldrich M4655) containing 10% fetal bovine serum (FBS; 305 mOsm/kg; Corning 35–010-CV, lot. 35010109). Coding sequences of target proteins were inserted downstream of the CAG promoter of the pEB6CAGMCS a/p vector and the resultant constructs were transfected into HEp-2 cells using Lipofectamine LTX & Plus Reagent (Invitrogen). Transfected cells were selected by 4-μg/ml puromycin treatment over 7 d. Cells were seeded at a density 4.0 × 10^4^ cells in 24-well plates 24 h before stress exposure. For exposure to hyperosmotic stress, the medium was replaced with freshly prepared hyperosmotic EMEM containing 10% FBS and various amounts of additional sucrose (final 100–350 mM; theoretical upshifts of osmolality are 100–350 mOsm/L) and cells were incubated for 48 h. After hyperosmotic exposure, the metabolic activities of treated cells were measured by replacing the medium with serum-free Opti-MEM (280–320 mOsm/kg, Invitrogen) containing the cell viability indicator PrestoBlue (Molecular Probes). PrestoBlue is a cell permeable resazurin-based reagent, which is converted to highly fluorescent resorufin by reducing power of living cells and thus metabolic activities can be quantitatively measured by detecting fluorescence. After incubation for 30 min, fluorescent signals were measured as an indication of cell viability with a Gemini EM Microplate Reader (Molecular Devices). Independent three wells were examined for each condition and relative cell metabolic activity was calculated by dividing each value by the mean value of the same cell strain at 0 mM additional sucrose. Improvements of cell viability were statistically analysed using one-sided upper *Dunnett*’s test against HEp-2 control cells.

## Results

### Identification of a putative mitochondrial LEA protein, RvLEAM, in *R. varieornatus*

**Fig 1:**
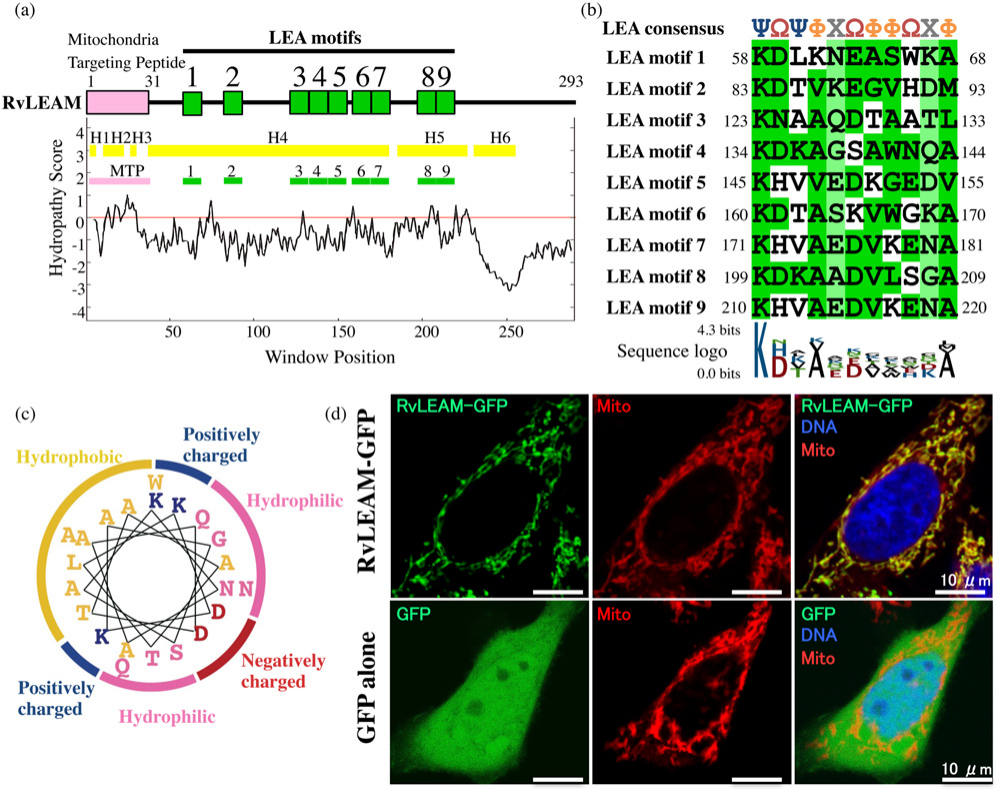
Protein structure and mitochondria-targeting potential of RvLEAM. (a) Schematic representation of RvLEAM protein structure with hydropathy plot. Pink box indicates Mitochondria Targeting Peptide (MTP) at the N-terminus and the nine green boxes indicate LEA motifs found in RvLEAM protein. The six yellow bars in the hydropathy plot indicate predicted helix regions (H1–6) (b) Alignment of LEA motif sequences of RvLEAM with consensus. Greek characters in the consensus sequence indicate the following: Ψ, basic residue; Ω, acidic or amide residue; Φ, aliphatic residue; X, non-conserved residue. Residues consistent with the consensus are highlighted in deep green, and amino acids at non-conserved positions are shown in light green. Sequence logo created from nine LEA motifs of RvLEAM is shown below the alignment. (c) Amphipathic distribution of amino acids in predicted helical region of repeating LEA motifs 3 and 4. Amino acids are indicated as hydrophilic (pink), negatively-charged (red), positively-charged (blue), and hydrophobic (yellow) residues. (d) Subcellular localization of RvLEAM-GFP fusion protein (top) and GFP alone (bottom) in human HEp-2 cells. GFP signals (left). Mitochondria stained with Mito-Tracker (centre). Merged images (right).

As candidate proteins involved in protecting mitochondria from desiccation stress, we detected an LEA protein potentially targeted to the mitochondria from our transcriptome database of *R. varieornatus*. We designated this protein RvLEAM. RvLEAM showed sequence similarity with nematode LEA proteins in the BLAST search (e-value < 1e-05) and matched with LEA-RELATED motif (PTHR23241) of PANTHER database in InterProScan search. Motif discovery program MEME and manual inspection revealed 9 LEA-like 11-mer motifs in the RvLEAM sequence (Fig. 1a-b, S1 Fig.). Seven of them formed three regions composed of contiguously repeated LEA-like motifs (Fig. 1a). Contiguous repeats of the 11mer LEA motif is characteristics of group 3 LEA proteins and its consensus sequence, ΨΩΨΦXΩΦΦΩXΦ [34], matched well with all motifs found in RvLEAM (Fig. 1b; Ψ, basic residue; Ω, acidic or amide residue; Φ, aliphatic residue; X, non-conserved residue). Similar to most LEA proteins, RvLEAM is highly hydrophilic (GRAVY score, -0.94) and a hydropathy plot showed no clear hydrophobic region over the entire sequence, suggesting that RvLEAM localized in the mitochondrial matrix rather than integrating into the mitochondrial membrane (Fig. 1a). Another characteristic of group 3 LEA proteins is their amphipathic helical structure under water-deficient conditions. The secondary structure prediction program predicted long helical regions that contained nine LEA-like motifs, and, in particular, a region containing two contiguously repeating LEA motifs (3 and 4) showed a typical amino-acid distribution forming putative amphipathic helix like LEA proteins (Fig. 1a, 1c).

We tested three subcellular localization prediction programs, TargetP, WoLF PSORT, and MitoProt2, and all three programs predicted that the RvLEAM protein localized in the mitochondria with high scores (S1 Table). TargetP also predicted a 31-mer mitochondria-targeting peptide at the N-terminus (Fig. 1a, S1 Fig.). To experimentally examine the mitochondrial-targeting potential of RvLEAM, we analysed the subcellular localization of RvLEAM in cultured mammalian cells by fusing GFP at the C-terminus. RvLEAM-GFP showed condensed localization in cells that was well co-localized with mitochondria stained by MitoTracker Red CMXRos (Fig. 1d). GFP alone was dispersed throughout cytoplasm, indicating that the mitochondrial localization of RvLEAM-GFP was due to the targeting potential of RvLEAM (Fig. 1d). Together, these findings suggested that RvLEAM is a mitochondria-targeted LEA protein, the first report of such a protein in tardigrades.

### RvLEAM protein localized in tardigrade mitochondria

**Fig 2:**
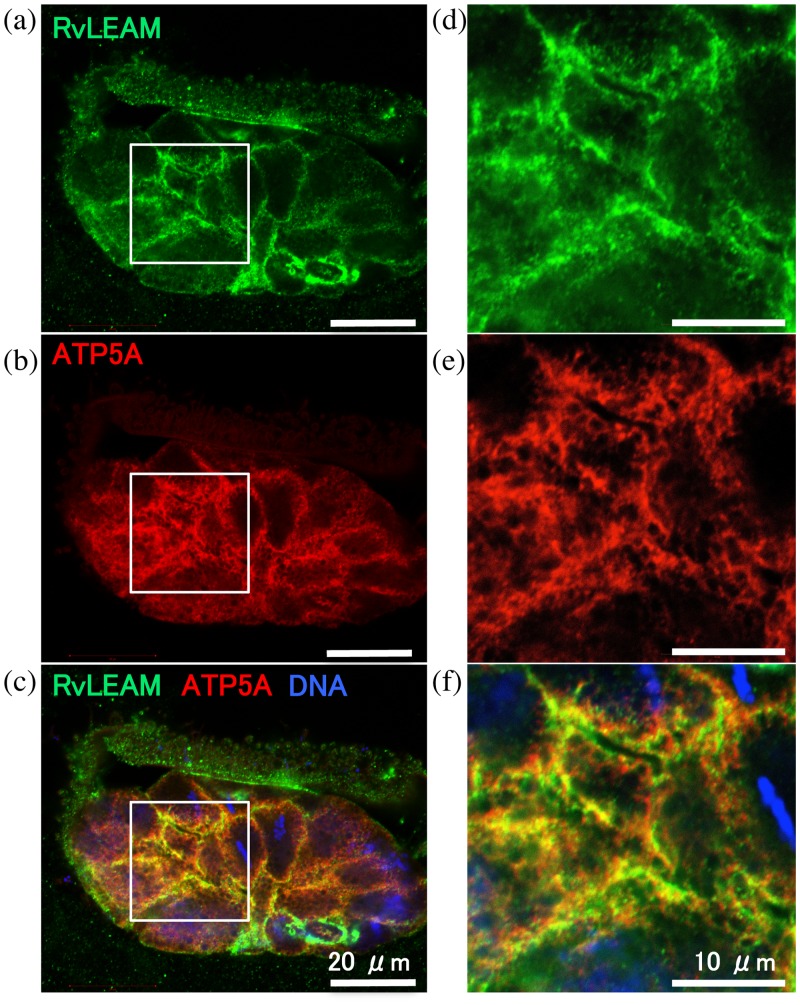
Mitochondrial localization of RvLEAM protein in tardigrade embryo. Tardigrade embryos were collected with eggshells and immunohistochemical analysis was performed using 10-μm frozen sections. Images show a representative section that was simultaneously reacted with anti-RvLEAM antiserum (a, d) and anti-ATP5A (mitochondrial ATP synthase) antibody (b, e). (c, f) Merged images. (d-f) Magnified images corresponding to the white boxes in (a-c).

To clarify the intact subcellular localization of RvLEAM in tardigrade cells, we performed immunohistochemical analysis using frozen sections of tardigrades. To clearly visualize subcellular localization, we chose early embryonic stage because embryonic cells are much larger than adult cells and the embryos also possess anhydrobiotic ability as adults do [24]. The sections of the tardigrade embryos were reacted with newly raised antiserum against RvLEAM. For simultaneous visualization of the mitochondria, the sections were co-stained with anti-ATP synthase (ATP5A) antibody as a mitochondrial marker. The fluorescent signals of RvLEAM were detected in almost all cells and their localization was mostly merged with those of ATP5A, indicating mitochondrial localization of RvLEAM in tardigrade cells (Fig. 2). In sections reacted with control antibodies, no signals were detected inside the eggshells (S2 Fig.), confirming the specific staining of RvLEAM and ATP5A in Fig. 2. This is the first immunohistochemical evidence for organelle-selective localization of identified LEA proteins in tardigrades. These findings are consistent with the RvLEAM-GFP localization in human cells (Fig. 1d) and validate the subcellular localization analysis using GFP-fusion protein in mammalian cells for tardigrade proteins. Tardigrades likely share at least a partially common mechanism with human cells to deliver proteins to the mitochondria.

### Heat-solubility of RvLEAM protein

**Fig 3:**
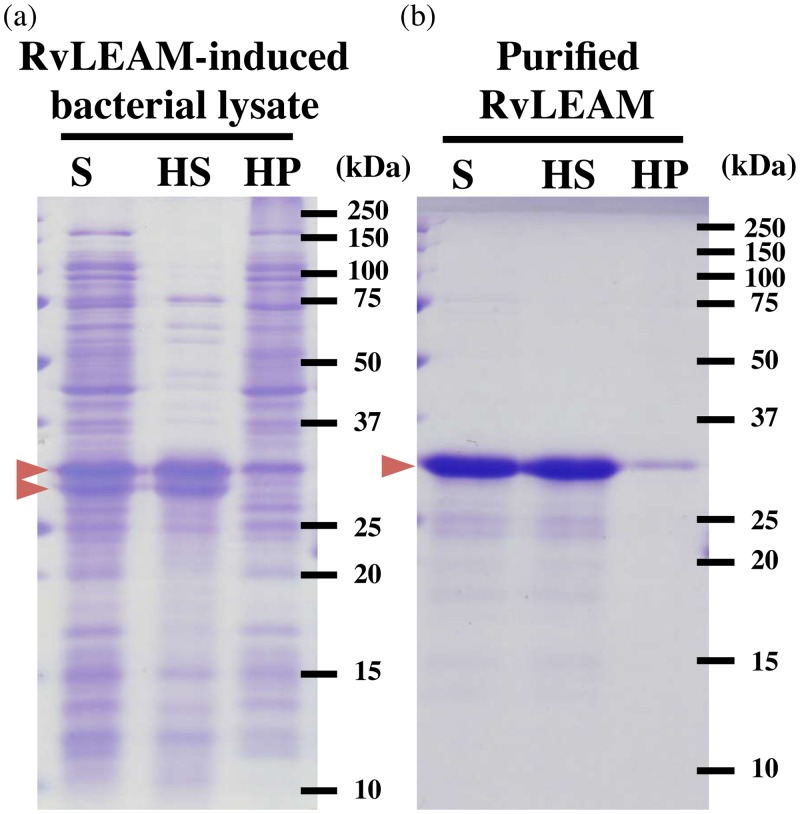
Heat-soluble property of RvLEAM protein. Heat-solubility of RvLEAM protein is shown using either RvLEAM-induced bacterial lysate (a) or purified RvLEAM protein (b). ‘S’ indicates starting sample before heat treatment. After heat treatment, the proteins were separated into a soluble fraction (HS) and insoluble precipitate (HP). Arrows indicate RvLEAM protein.

LEA proteins are highly hydrophilic and maintain their solubility even after heat treatment. To determine whether RvLEAM has biochemical properties similar to those of other LEA proteins, we examined the heat-solubility of RvLEAM protein. RvLEAM protein was bacterially expressed and subjected to heat treatment as either a bacterial lysate or purified protein solution. Heat treatment of the bacterial lysate resulted in the precipitation of most bacterial proteins, whereas RvLEAM protein was mostly recovered in the supernatant even after heat treatment (Fig. 3a), indicating a heat-soluble property of RvLEAM proteins. Furthermore, we confirmed that purified RvLEAM protein alone exhibited heat-solubility (Fig. 3b), indicating that the heat-solubility of RvLEAM is a characteristic of the protein itself and requires no other macromolecules like sugars or polyamines. These results confirmed that RvLEAM protein belongs to the LEA protein family based on both its primary sequence and biochemical properties.

### MAHS, a novel mitochondrial heat-soluble protein unique to tardigrades

**Fig 4:**
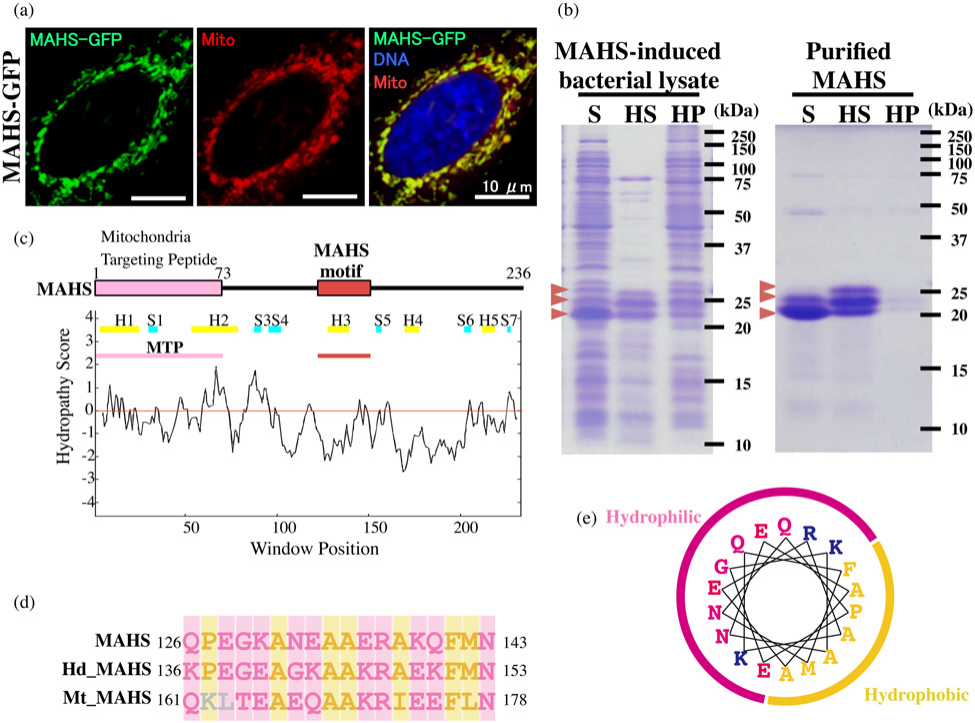
Subcellular localization of a novel mitochondrial heat-soluble MAHS protein. (a) Subcellular localization of MAHS-GFP fusion protein in human HEp-2 cells. (b) Heat-solubility of MAHS protein using bacterial lysate (left) or purified protein (right). Starting samples before heat treatment (S), soluble fraction after heat treatment (HS), and insoluble precipitate (HP) are shown. Red arrows indicate MAHS protein. (c) MAHS protein structure with hydrophathy plot. Mitochondria Targeting Peptide and conserved MAHS motif are indicated by the pink box and red box, respectively. Yellow and blue bars in the hydropathy plot indicate predicted helix regions (H1–5) and strand regions (S1–7), respectively. (d) Sequence alignment of conserved MAHS motifs among three tardigrade species, *R. varieornatus*, *H. dujardini* (Hd), and *M. tardigradum* (Mt). Pink and yellow indicate hydrophilic and hydrophobic residues, respectively (d) Amphipathic nature of predicted helix in MAHS motif. Amino acids are coloured as described in Fig. 1c.

A previous heat-soluble proteome study revealed that the most abundant heat-soluble proteins in tardigrade are not LEA proteins, but tardigrade-unique proteins, such as CAHS and SAHS proteins [23]. Neither CAHS nor SAHS is localized in the mitochondria, and RvLEAM is currently the only known mitochondrial heat-soluble protein in tardigrades. LEA proteins work synergistically with trehalose to protect macromolecules or cells from water stress [17,35–37]. In tardigrades, however, there is low or no accumulation of trehalose in the dehydrated state [8], and thus there could be other protective molecules in addition to LEA proteins in tardigrades. Tardigrade-unique heat soluble proteins, such as CAHS and SAHS proteins, are possible candidates. In analogy with CAHS and SAHS proteins, we postulated that there could be novel tardigrade-unique heat-soluble proteins in the mitochondria. To evaluate this possibility, we identified six genes from abundantly expressed genes in our transcriptome data that are unique to tardigrades and are predicted to localize in the mitochondria. We examined the mitochondria-targeting potential of these candidate gene products by expressing GFP-fused protein in mammalian culture cells. Two of them, MAHS and ATPM1, showed mitochondrial localization (Fig. 4a, S3 Fig.). Comparison of the localization pattern of GFP-fusion protein with predicted score values of the three prediction programs showed that MitoProt2 provided good estimates regarding mitochondrial localization of the examined proteins (S1 Table). MitoProt2 would be a suitable bioinformatic tool for predicting mitochondrial localization for the tardigrade proteome in future studies. A heat-solubility assay of these two proteins demonstrated that only one protein had a heat-soluble property similar to that of RvLEAM protein, either in bacterial lysate or as a purified protein (Fig. 4b, S3b Fig.). We designated this protein MAHS protein in analogy with CAHS and SAHS proteins. Although purified MAHS protein was recovered in the soluble fraction after heat treatment, the heat-treated MAHS protein showed relatively slower migration in sodium dodecyl sulphate-polyacrylamide gel electrophoresis (Fig. 4b), indicating a possible conformational change induced by heat treatment.

The MAHS protein contained a predicted long mitochondrial targeting peptide at the N-terminus and the resultant putative mature form was highly hydrophilic (GRAVY score of-0.77) like RvLEAM protein (Fig. 4c). A BLASTP search in non-redundant (nr) database retrieved no sequences, and TBLASTN searches in EST/TSA databases retrieved only two sequences of other tardigrades (e-value < 1); one from the EST database of *Hypsibius dujardini* and the other from the TSA database of *Milnesium tardigradum*. No LEA proteins were retrieved in the search and also no LEA-like motif was found by either a InterProScan search or manual inspection. These findings suggested that the MAHS protein forms a novel heat-soluble protein family targeted to the mitochondria, which is unique to and conserved in tardigrades. Sequence comparison among putative tardigrade MAHS proteins revealed a conserved region in the middle of the protein (S4 Fig.), and this region was partially predicted to form an alpha-helix by PORTER predication software (Fig. 4c-d), potentially with an amphipathic property (Fig. 4e), implying that MAHS proteins have a role similar to that of LEA proteins in anhydrobiosis.

### RvLEAM and MAHS proteins improved osmotic tolerance of human cells

**Fig 5:**
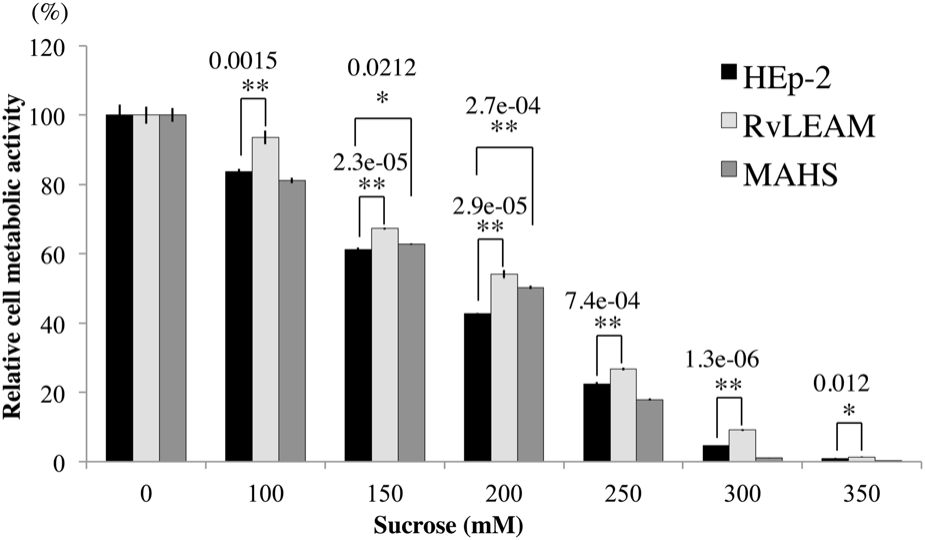
Effects of RvLEAM and MAHS proteins on metabolic activity of human cells under hyperosmotic stress. Human HEp-2 cells (black), and HEp-2 cells expressing RvLEAM (light grey) or MAHS (dark grey) were exposed to medium supplemented with defined concentrations of sucrose for 48 h. After replacement of the medium with serum-free isotonic medium, cell metabolic activities were analysed using PrestoBlue reagents. Metabolic activities at each sucrose concentration are shown as a percentage of the mean activity observed in the cells at 0 mM sucrose (n = 3, ±SEM). Statistical significance against untransfected HEp-2 cells was determined by *Dunnett*’s test (*, *P*<0.05; **, *P*<0.01). *P*-values are shown above asterisks where statistically significant.

LEA proteins are thought to play an important role in protecting macromolecules from desiccation stress in anhydrobiotic animals [9]. Introduction of LEA proteins to non-anhydrobiotic animal cells, like human cells, improved cellular viability and cellular integrity in water-deficient conditions such as dehydration or hyperosmotic treatment [16–18]. In these assays, the hyperosmotic treatment was relatively mild water stress compared to dehydration, enabling more sensitive detection of the protection ability of the introduced genes. In accordance, we tested whether the tardigrade mitochondrial heat-soluble proteins also improve the tolerability of human cells against hyperosmotic stress. First, we introduced RvLEAM or MAHS to human HEp-2 cells and established stably transfected cells that constitutively expressed either RvLEAM or MAHS under the control of the CAG promoter. The cells were shocked by exposure to hyperosmotic medium containing supplemented sucrose (final 100 mM to 350 mM) for 2 d, and the retained metabolic activities were assessed by measuring reducing power of cells using cell viability indicator in isotonic medium. Cells expressing RvLEAM had significantly higher metabolic activities, compared to untransfected control cells, at all examined sucrose concentrations (Fig. 5). Metabolic activity was increased two-fold by RvLEAM at 300 mM sucrose and exceeded that of untransfected cells by at least 10% in any examined condition. In addition, cells expressing MAHS also had significantly increased metabolic activities at 150 mM and 200 mM sucrose. The best improvement by MAHS (~20%) was observed at 200 mM sucrose, which is close to the EC50 value (179 mM) of untransfected cells (Fig. 5). The results suggested that mitochondrial heat-soluble proteins of tardigrades, even non-LEA protein like MAHS, improve the tolerability of human cells to hyperosmotic stress.

## Discussion

In the present study, we identified two novel heat-soluble proteins targeted to mitochondria, RvLEAM and MAHS, from the anhydrobiotic tardigrade *R. varieornatus* (Figs. 1, 4). RvLEAM is a group 3 LEA protein family member, whereas MAHS protein shows no sequence similarity to known protein families, including LEA proteins, nor known motifs. MAHS—like sequences were found in EST/TSA databases of other tardigrades, indicating that MAHS protein comprises a novel heat-soluble protein family conserved in tardigrades (Fig. 4). Despite differences in the primary structures between MAHS and RvLEAM, both proteins are highly hydrophilic and contain potential amphipathic helical regions (Figs. 1, 4). Furthermore, transfection of RvLEAM or MAHS improved cell viability against hyperosmotic stress in cultured human cells (Fig. 5). In LEA proteins, the amphipathic helix is suggested to be important for loose interactions with other macromolecules to prevent undesirable aggregation or conformational changes of proteins and liposomes, so-called ‘molecular shielding’ [38,39]. In MAHS protein, an apparently conserved region, the MAHS motif, has the potential to form an amphipathic helix (Fig. 4), consistent with the probably protective role of the amphipathic helix.

Introduction of RvLEAM improved the metabolic activity of human cells 2-fold (100% increase) at 300 mM sucrose (upshift of ~300 mOsm). Two studies have examined the effects of animal LEA proteins to improve osmotic tolerance in non-anhydrobiotic animal cells. In one study, a cytosolic LEA protein of the anhydrobiotic nematode transfected to human T-REx293 cells improved metabolic activity by 20% to 70% compared to uninduced cells after 2-d exposure to hyperosmotic medium (400 mOsm upshift with various solutes) [16]. In the second study, mitochondrial LEA protein of the anhydrobiotic crustacean Artemia improved the membrane integrity of insect cells by 20% to 60% in 50 to 200 mM sucrose. Although there are many differences in the cell types, hyperosmotic solutes, and methods used to measure cellular viability, improvements by RvLEAM were comparable to, or even better than, those by other animal LEA proteins in previous reports. Thus, the newly identified tardigrade mitochondrial LEA protein, RvLEAM, also improves the stress tolerability of sensitive cells. Introduction of MAHS protein induced significant improvements in the hyperosmotic tolerance of human cells (Fig. 5). The improvement, however, was only observed within a particular osmotic range of 150 to 200 mM sucrose (upshift of 150~200 mOsm) with up to a 20% increase. In tardigrades, both heat soluble proteins, MAHS and RvLEAM, likely localize in the mitochondria. MAHS protein might require other molecules, like RvLEAM, for efficient protection, as MAHS protein alone provided significant, but comparatively less, improvement of cellular activity in the hyperosmotic condition.

It is unclear why tardigrades contain at least two types of mitochondrial heat-soluble proteins. A possible explanation is that MAHS and RvLEAM protect different types of macromolecules; for example, one may protect proteins and the other lipids. The anhydrobiotic rotifer *Adineta ricciae* has two LEA proteins, ArLEA1A and ArLEA1B [15]. While ArLEA1A protects proteins from undesirable aggregation, ArLEA1B enhances protein aggregation, interacts with lipids preferentially, and is potentially involved in membrane protection. This could also be the case for RvLEAM and MAHS proteins. Another possible explanation is that simultaneous expression of two proteins contributes to increase the availability of heat-soluble proteins in mitochondria. Identification of the targets of RvLEAM and MAHS in future studies will be useful for distinguishing between these two explanations.

Based on the mitochondria-selective localization of RvLEAM and MAHS proteins (Figs. 1d, 2, 4a), they likely protect macromolecules inside the mitochondria rather than the whole cell content. How RvLEAM and MAHS proteins confer osmotic tolerance to cells, however, remains mysterious. Mitochondria are the energy centre of eukaryotic cells and produce ATP through oxidative phosphorylation, which simultaneously generates reactive oxygen species (ROS), and thus mitochondria are a major production source of ROS. In normal environments, ROS detoxification mechanisms in mitochondria are sufficient to counteract the generated ROS [40]. Water stress enhances ROS accumulation [41–43], however, which could exceed the detoxification capacity of mitochondria under stressed conditions. Thus, mitochondrial protection against ROS could be important for cellular survival. In addition, mitochondrial dysfunction induces apoptosis by releasing cytochrome C to the cytoplasm [40,44,45]. Therefore, mitochondrial protection could be important to protect cells against apoptosis. Our results, in conjunction with reported similar improvements by the mitochondrial LEA protein of Artemia [18], suggest that the preservation of mitochondrial integrity is important and partly sufficient to afford cellular tolerability against water stress. Simultaneous introduction of molecules that protect other cellular compartments could further enhance tolerability.

In our previous heat-soluble proteome analysis, we identified two tardigrade-unique heat-soluble protein families, CAHS and SAHS, as predominant heat-soluble proteins in the anhydrobiotic tardigrade *R. varieornatus*. Neither MAHS nor RvLEAM was detected in the previous study, possibly due to the small proportion of mitochondria in the tardigrade lysate, and thus other proteins in the cytoplasm and/or extracellular space might overwhelm MAHS and RvLEAM. To date, three tardigrade-unique heat-soluble protein families have been identified, MAHS, CAHS, and SAHS. Their subcellular localizations are mutually exclusive and together they cover most cellular components: MAHS in the mitochondria, CAHS in the cytoplasm and nucleus, and SAHS in the extracellular space and secretory organelles.

These tardigrade-unique heat-soluble proteins are hydrophilic and have biochemical properties similar to LEA proteins, suggesting their involvement in tolerability, but experimental evidence was lacking. The present study provides the first evidence that newly identified MAHS, a tardigrade-unique heat-soluble protein, confers tolerability against water stress. This is the first report of the protective effect of tardigrade-unique heat-soluble proteins.

Why have tardigrades evolved to utilize such unique heat-soluble protein complements alongside LEA proteins, unlike other anhydrobiotic animals? The difference in trehalose accumulation might be a clue to answering this question. Anhydrobiotic arthropods and nematodes in a dehydrated state accumulate large amounts of trehalose [4–6]. In contrast, there is no or low accumulation of trehalose in anhydrobiotic tardigrades [8], thus an alternative protective mechanism/molecule might be required. Tardigrade-unique heat-soluble proteins, including MAHS identified in this study, are candidate protective proteins that complement the small amount of trehalose present.

We identified the first mitochondrial complement of tardigrade-unique heat-soluble proteins and demonstrated its protective activity. The current repertoire of tardigrade-unique heat-soluble proteins covers various cellular components, including mitochondria, which seem to be key target organelles to confer protection to cells against water stress. This repertoire of proteins provides clues to help elucidate the protection mechanism of tardigrades against stress, and may contribute to the development of a method to confer more robust tolerability to stress-sensitive cells of other species, including humans.

## Supporting Information

S1 Fig Multiple alignments of LEAM proteins among tardigrade species. (TIF)

S2 Fig Specific reactivity of anti-RvLEAM antiserum and anti-ATP5A antibody in immunohistochemistry. (TIF)

S3 Fig Characteristics of predicted tardigrade-unique proteins in mitochondria. (TIF)

S4 Fig Multiple alignments of MAHS proteins among tardigrade species. (TIF)

S1 Table Subcellular localization of potential mitochondria-targeted LEA protein and tardigrade-unique proteins. (TIF)
