# Supplementary figures and images for: Novel Mitochondria-Targeted Heat-Soluble Proteins Identified in the Anhydrobiotic Tardigrade Improve Osmotic Tolerance of Human Cells
Source: PLoS One. 2015 Feb 12;10(2):e0118272. doi: 10.1371/journal.pone.0118272 (PMC4326354; doi:10.1371/journal.pone.0118272)

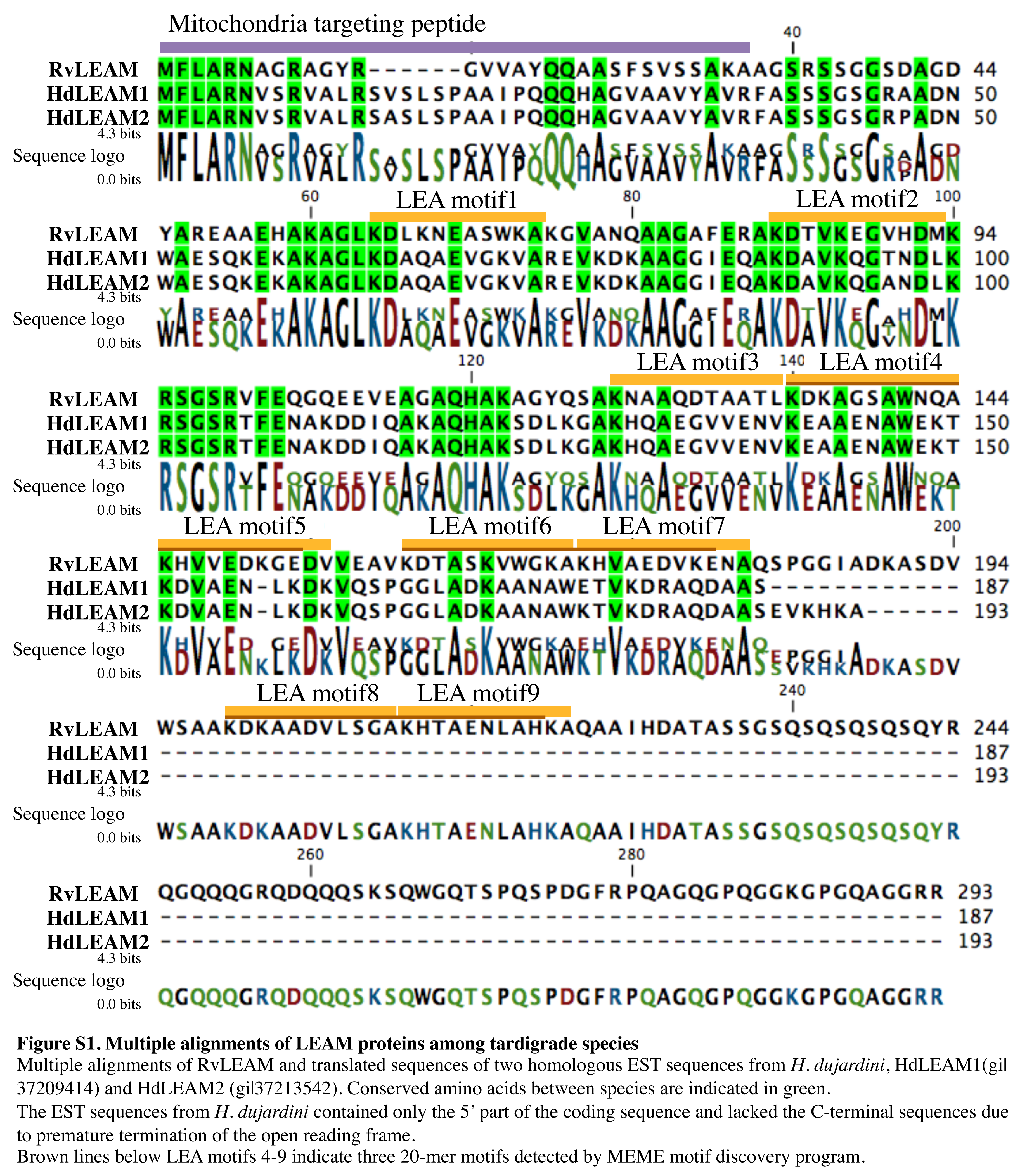

Supplement: S1 Fig — (TIF) [file pone.0118272.s001.tif]

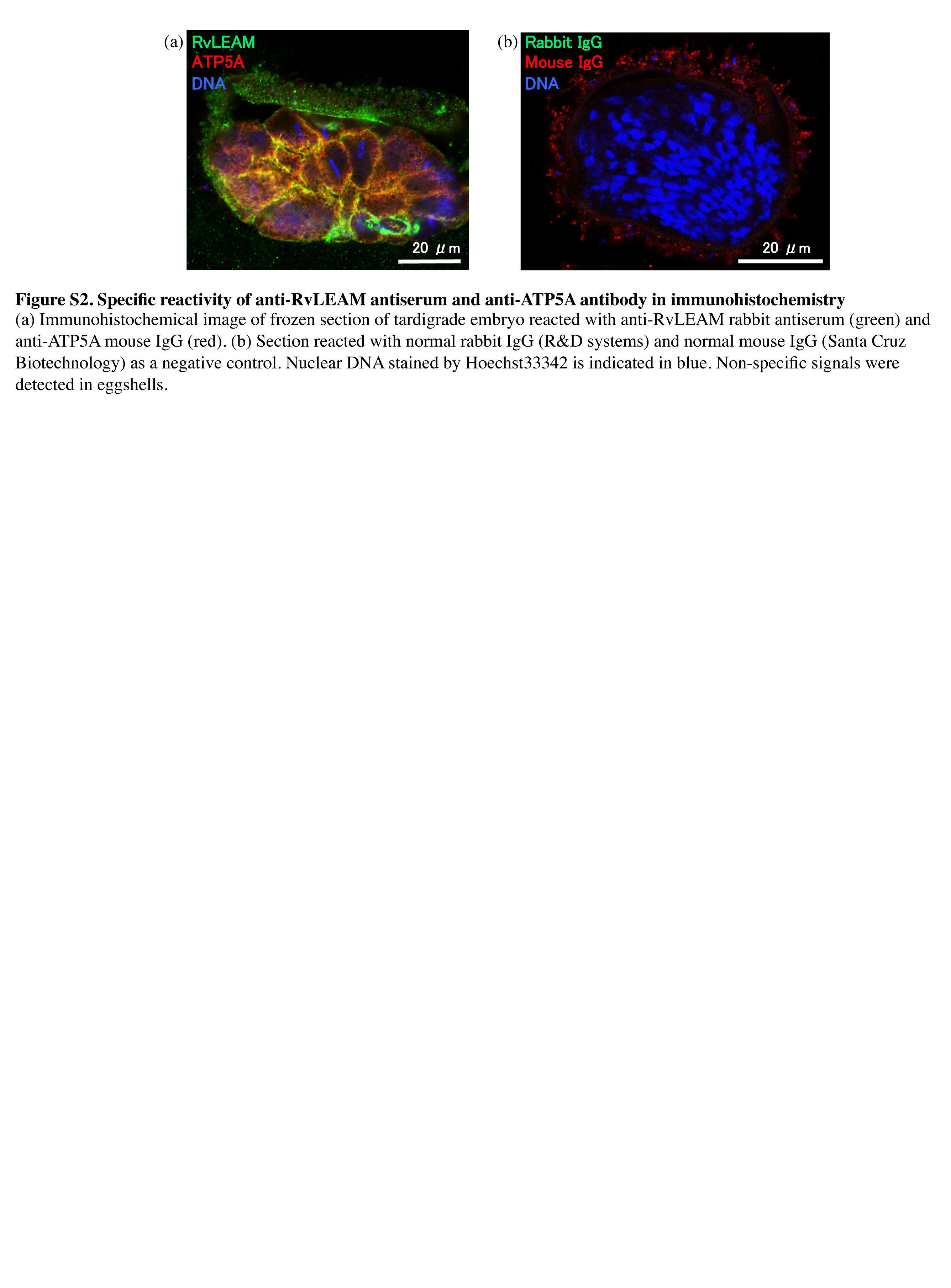

Supplement: S2 Fig — (TIF) [file pone.0118272.s002.tif]

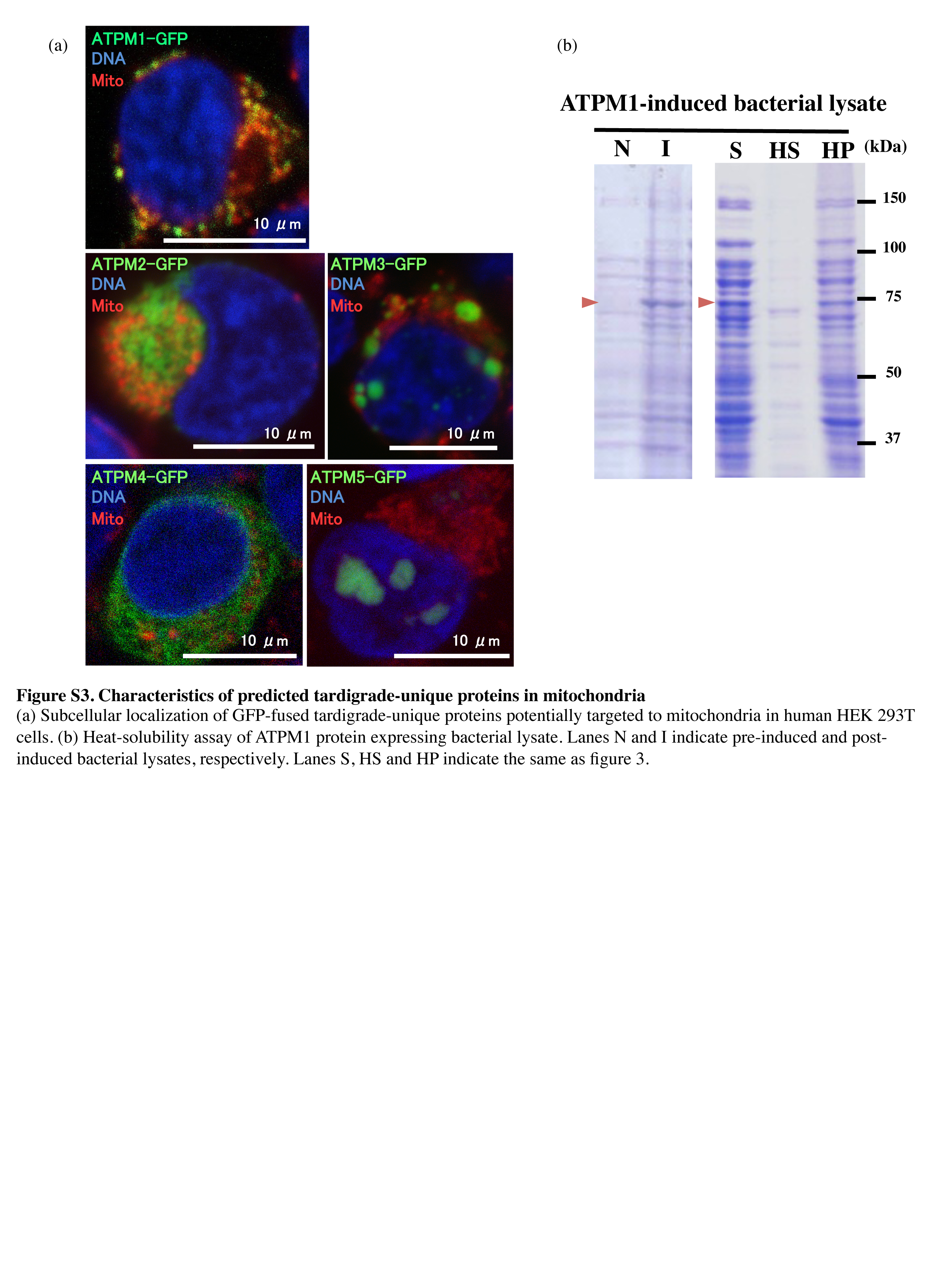

Supplement: S3 Fig — (TIF) [file pone.0118272.s003.tif]

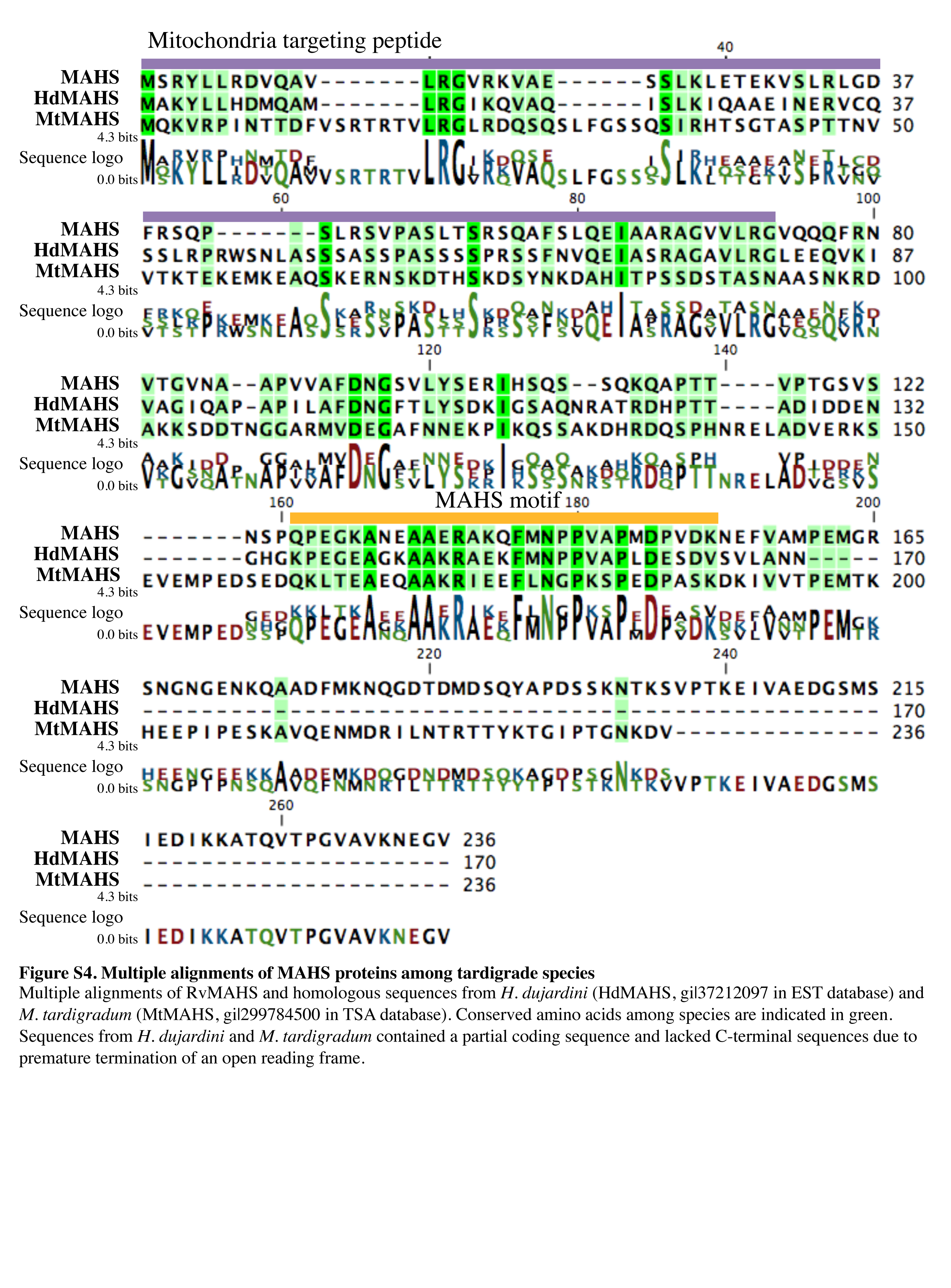

Supplement: S4 Fig — (TIF) [file pone.0118272.s004.tif]

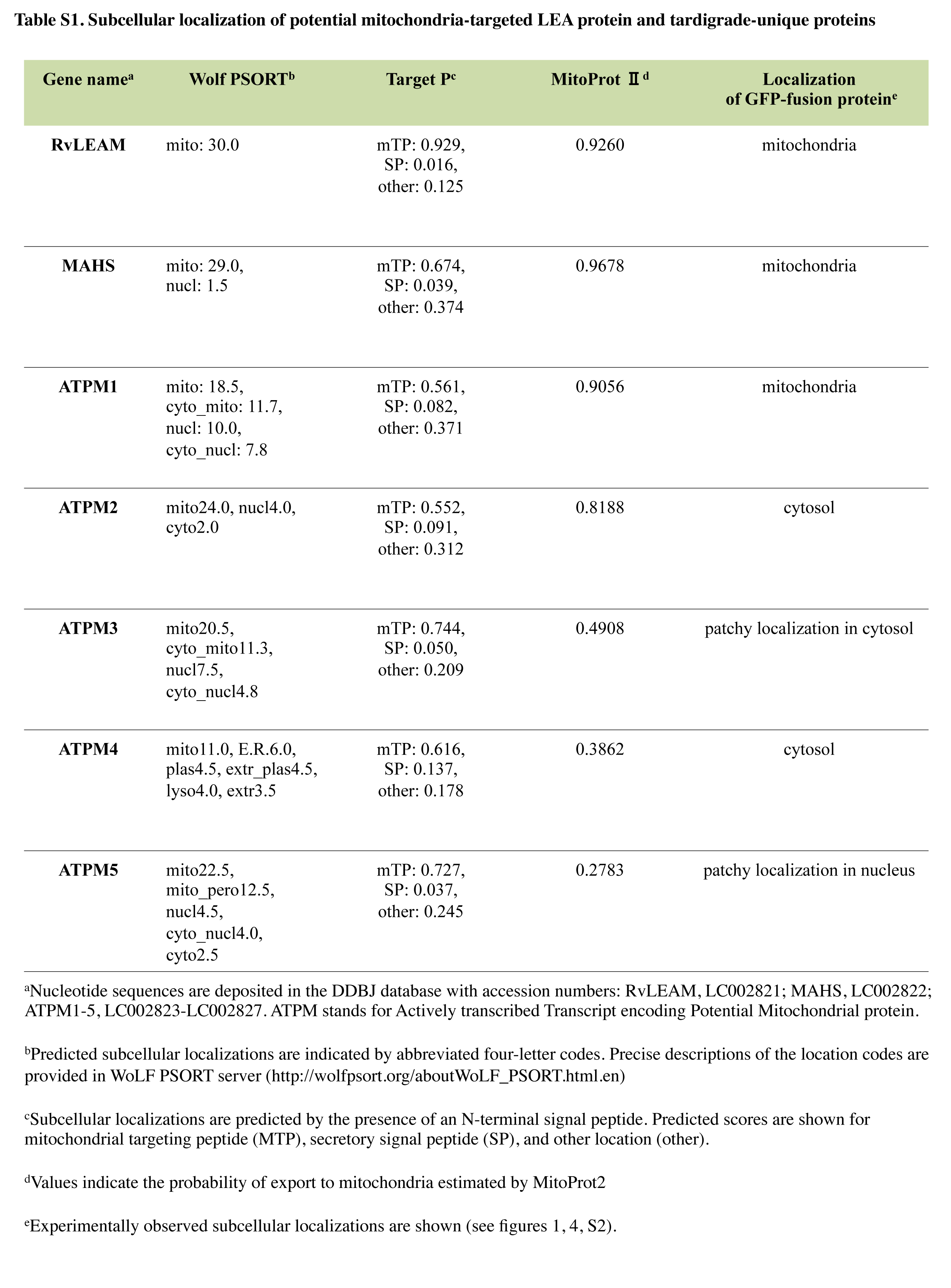

Supplement: S1 Table — (TIF) [file pone.0118272.s005.tif]
